# Cognitive impairment and depressive symptoms to predict renal outcome and mortality in older adult patients

**DOI:** 10.1371/journal.pone.0342924

**Published:** 2026-03-02

**Authors:** Bokeung Peun, Ho Jun Chin, Jung-Yeon Choi, Kwang-il Kim, Heeseung Choi

**Affiliations:** 1 Department of Psychiatric Nursing, College of Nursing, Seoul National University, Seoul, Korea; 2 Department of Internal Medicine, Seoul National University College of Medicine, Seoul, Korea; 3 Department of Internal Medicine, Seoul National University Bundang Hospital, Seong-Nam, Korea; 4 Seoul National University College of Nursing and Research Institute of Nursing Science, Seoul, Republic of South Korea; Azienda Ospedaliero Universitaria Careggi, ITALY

## Abstract

Cognitive impairment and depressive symptoms are common in older adults, but their impacts on adverse renal and survival outcomes remain unclear. This retrospective cohort study examined associations of cognitive impairment and depressive symptoms with renal replacement therapy (RRT) initiation and mortality among 5,191 adults aged ≥ 65 years. Cognitive impairment and depressive symptoms were assessed using the Korean Mini-Mental State Examination (MMSE-KC) and the Korean Short Geriatric Depression Scale (SGDS-K), respectively. During a mean follow-up of 45 months, 1,512 participants (29.1%) died and 68 (1.3%) initiated RRT. Depressive symptoms (SGDS-K score ≥ 5) were associated with increased risks of both incident RRT (HR 2.066, p = 0.004) and mortality (HR 1.565, p < 0.001). Cognitive impairment (MMSE-KC score ≤ 23) was associated with both incident RRT (HR 2.028, p = 0.007) and mortality (HR 1.728, p < 0.001). Subgroup analyses demonstrated that depressive symptoms remained associated with RRT risk across age groups and renal function strata, while cognitive impairment was linked to RRT primarily among those with preserved renal function or age < 75 years. Both factors were consistently associated with higher mortality across subgroups. Although receiver operating characteristic curve analysis showed comparable predictive value for RRT, cognitive impairment demonstrated greater discriminative ability for mortality. These findings suggest that depressive symptoms may serve as a relevant marker of renal deterioration requiring RRT, whereas cognitive impairment may more closely reflect mortality risk. Routine psychological and cognitive assessments may support early identification of high-risk patients and inform prevention strategies in geriatric care.

## Introduction

Cognitive impairment and depressive symptoms are prevalent among older adults and have been linked to various adverse health outcomes [1,2]. Moreover, as aging progresses, cognitive decline and mood disorders contribute to frailty, decreased independence in daily activities, and greater reliance on healthcare services [3,4]. These conditions also affect older adults’ physical health, leading to lower treatment adherence, increased hospitalization rates, and a higher mortality risk [5,6]. Cognitive impairment and depressive symptoms have also been associated with prolonged hospitalization and increased mortality [7] and overall health decline [8,9]. Specifically, while cognitive impairment may hinder symptom recognition and treatment adherence [10], depressive symptoms can increase systemic inflammation and cardiovascular risk, contributing to functional decline [11,12]. Meanwhile, the direct impact of cognitive impairment and depressive symptoms on long-term outcomes, such as mortality and advanced medical interventions, remains unclear.

Thus, this study examined whether cognitive impairment and depressive symptoms are independently associated with mortality and the need for intensive medical interventions, such as renal replacement therapy (RRT), in older adults. Using a large geriatric cohort, we assessed the persistence of these associations after adjusting for comorbid conditions. A clearer understanding of these relationships may help inform early screening and intervention strategies, ultimately supporting better health outcomes and quality of life in aging populations.

## Subjects and methods

### Study design and population

**Fig 1:**
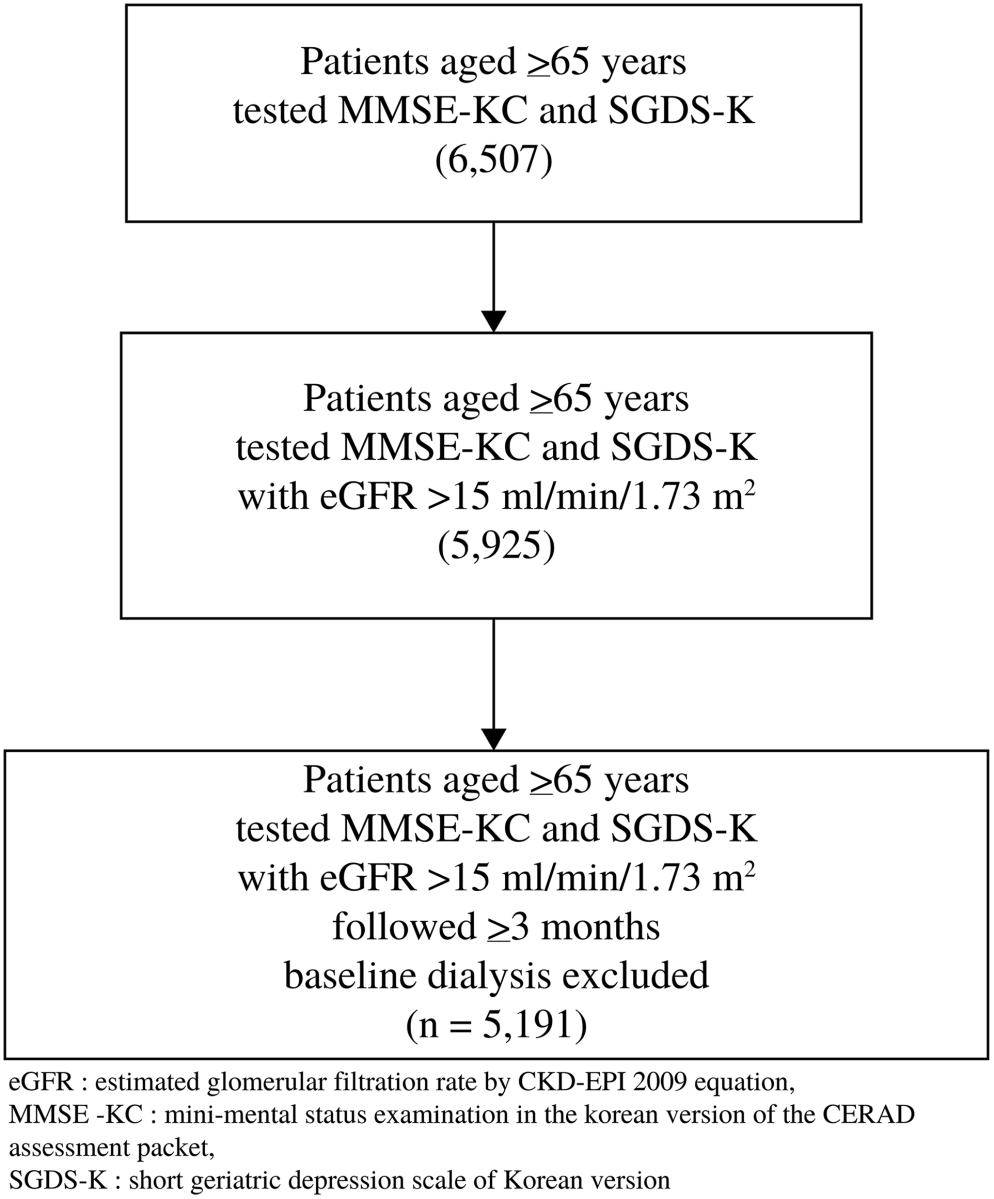
Flow chart of participant selection.

This retrospective cohort study included 5,191 individuals aged ≥ 65 years who underwent geriatric assessment at a university hospital in Korea between January 2016 and December 2020. To ensure inclusion across a broad range of kidney function levels, participants had to have an estimated glomerular filtration rate (eGFR) >15 ml/min/1.73 m^2^ at baseline and at least three months of follow-up. Individuals with pre-existing end-stage renal disease requiring dialysis at baseline were excluded to allow the study to focus on incident RRT events (Fig 1). Clinical data, including initiation of RRT, were extracted from electronic health records. Mortality data were obtained from the nationwide death registry maintained by the Ministry of the Interior and Safety of Korea, and deterministically linked to hospital electronic health records using unique personal identifiers, ensuring complete mortality follow-up for all Korean participants.

This study was approved by the Institutional Review Board of Seoul National University Bundang Hospital (IRB No. B-2406-909-105). Data extraction and analysis were performed after approval was granted, with permission for the retrospective use of anonymized clinical data (IRB approval: June 5, 2024; continuing approval: April 28, 2025). The requirement for informed consent was waived owing to the retrospective nature of the study and the use of anonymized data. The completeness of the data is shown in S1 Table.

### Assessment of cognitive impairment and depressive symptoms

The comprehensive geriatric assessment (CGA) is a multidimensional, interdisciplinary diagnostic process designed to evaluate older adults’ overall health. It integrates frailty assessments, disease management, medication reviews, and rehabilitation assessments for a holistic and individualized approach to older adult care. To gain a comprehensive understanding of each patient’s health status, cognitive impairment and depressive symptoms were systematically evaluated as part of the comprehensive geriatric assessment.

Cognitive impairment was assessed using the Korean Mini-Mental State Examination (MMSE-KC), which consists of 19 items with a total score ranging from 0 to 30 and is a widely used tool for detecting cognitive impairment in older adults [13]. The MMSE-KC evaluates multiple cognitive domains, including orientation, memory registration, attention and calculation, recall, language, and visuospatial abilities, with a maximum possible score of 30 points. Based on established thresholds, a score of 23 or lower indicates cognitive impairment, 20–23 suggests mild impairment, and 19 or lower indicates moderate-to-severe impairment. This assessment was conducted as part of the comprehensive geriatric assessment during hospital admissions or outpatient visits.

Depressive symptoms were evaluated using the Korean Short Geriatric Depression Scale (SGDS-K), a validated screening tool for assessing depressive symptoms in older adults [14]. The SGDS-K consists of 15 yes/no items designed to minimize the influence of physical symptoms on depression assessment. A total score of less than 5 indicates no significant depressive symptoms, a score between 5–9 indicates mild depressive symptoms, and a score ≥ 10 indicates moderate-to-severe depressive symptoms. Similar to the cognitive assessment, the SGDS-K was administered as part of the comprehensive geriatric assessment during hospital admissions or outpatient visits.

### Outcome measures

The primary outcomes were all-cause mortality and incident initiation of RRT. Mortality data, including deaths from all causes, were obtained from hospital records and national death registries. RRT initiation was defined as the first occurrence of hemodialysis or peritoneal dialysis during the follow-up period.

### Statistical analysis

Baseline data on demographic and clinical characteristics (i.e., age, sex, hypertension, diabetes, cardiovascular disease, and cancer history) as well as laboratory parameters (e.g., hemoglobin, serum albumin, and eGFR) were analyzed using descriptive statistics, with continuous variables expressed as mean ± standard deviation (SD) and categorical variables as percentages. Differences between groups were analyzed using student’s *t*-test for continuous variables and Pearson’s chi-square test for categorical variables.

Cox proportional hazards regression models were used to examine the associations of cognitive impairment (MMSE-KC) and depressive symptoms (SGDS-K) with clinical outcomes. Hazard ratios (HRs) with 95% confidence intervals (CIs) were calculated to quantify the associations with incident RRT and mortality after adjusting for potential confounders. All covariates used in multivariable modeling were complete; therefore, complete-case analysis was applied without imputation.

Kaplan–Meier survival analysis was used to compare survival outcomes across cognitive impairment and depressive symptoms groups. Log-rank tests were conducted to assess the statistical significance of survival differences between the groups. Receiver operating characteristic (ROC) curve analysis and area under the curve (AUC) were used to evaluate the discriminative ability of MMSE-KC and SGDS-K for RRT initiation and mortality. Differences in AUC values between MMSE-KC and SGDS-K were tested for statistical significance.

To account for the competing risk of death when assessing incident RRT, we additionally performed Fine–Gray sub-distribution hazard models using Stata, treating death as a competing event. As a time-dependent measure of discriminative performance, we also calculated concordance indices (C-indices) from the Cox models. The proportional hazards assumption for Cox models was evaluated using Schoenfeld residuals (global and covariate-specific tests), and no meaningful violations were detected.

All statistical analyses were conducted using IBM SPSS Statistics version 29 (IBM Corp., Armonk, NY, USA) or Stata version 17 (StataCorp LLC, College Station, TX, USA), with the significance level set at a p < 0.05.

## Results

Of 5,191 participants, 2,945 were categorized according to cognitive impairment scores into CG1 (MMSE-KC scores ≥ 24), 1,191 into CG2 (MMSE-KC scores of 20–23), and 1,055 into CG3 (MMSE-KC scores ≤ 19). Similarly, participants were grouped according to depressive symptom scores into DG1 (SGDS-K scores <5), DG2 (SGDS-K scores of 5–9), and DG3 (SGDS-K scores ≥ 10).

During an average follow-up period of 45 months, 1,512 deaths (29.1%) and 68 cases (1.3%) of incident initiation of RRT were recorded. Participants with cognitive impairment (CG2–CG3; MMSE-KC scores ≤ 23) were significantly older (p < 0.001) and had a higher prevalence of cardiovascular disease, diabetes mellitus, and hypertension, but a lower prevalence of cancer (p < 0.001 for all) compared with participants in CG1 (MMSE-KC scores ≥ 24).

Similarly, participants with depressive symptoms (SGDS-K scores ≥ 5) were also older (p < 0.001) and had a higher prevalence of cardiovascular disease, diabetes mellitus, and hypertension (p < 0.001 for all), but a lower prevalence of cancer (p < 0.001) than those without depressive symptoms. Laboratory findings indicated that both cognitive impairment and depressive symptoms were associated with lower hemoglobin and albumin levels and eGFR (all p < 0.001; Table 1).

**Table 1:** Baseline characteristics of the study population by cognitive impairment and depressive symptoms.

	Cognitive impairment Group				Depression symptoms Group			
	Group 1 (CG1)	Group 2 (CG2)	Group 3 (CG3)	P-value	Group 1 (DG1)	Group 2 (DG2)	Group 3 (DG3)	P-value
Number (%)	2945 (56.7)	1191 (22.9)	1055 (20.3)		3220 (62.0)	1295 (24.9)	676 (13.0)	
Age (year)	76.7 ± 5.2	78.7 ± 5.5	80.7 ± 5.7	<0.001	77.5 ± 5.4	78.6 ± 5.9	78.8 ± 5.5	<0.001
Sex (male, %)	1624 (55.1)	459 (38.5)	305 (28.9)	<0.001	1632 (50.7)	526 (40.6)	230 (34.0)	<0.001
BMI (kg/m^2^)	24.1 ± 3.3	24.1 ± 3.7	23.6 ± 4.0	<0.001	24.2 ± 3.5	23.7 ± 3.8	23.6 ± 3.6	<0.001
SBP (mmHg)	127 ± 18	128 ± 18	127 ± 19	0.623	127 ± 18	127 ± 18	128 ± 18	0.362
DBP (mmHg)	81 ± 27	80 ± 27	80 ± 27	0.433	81 ± 26	81 ± 28	82 ± 27	0.468
DM (%)	554 (18.8)	266 (22.3)	280 (26.5)	<0.001	622 (19.3)	304 (23.5)	174 (25.7)	<0.001
HTN (%)	715 (24.3)	296 (24.9)	297 (28.2)	0.043	760 (23.6)	352 (27.2)	196 (29.0)	0.002
CVD (%)	347 (11.8)	138 (11.6)	176 (16.7)	<0.001	372 (11.6)	191 (14.7)	98 (14.5)	0.005
Cancer (%)	1723 (58.5)	682 (57.3)	517 (49.0)	<0.001	1908 (59.3)	678 (52.4)	336 (49.7)	<0.001
CMI (number)	2.38 ± 1.76	2.50 ± 1.81	2.55 ± 1.79	0.012	2.39 ± 1.70	2.51 ± 1.91	2.58 ± 1.84	0.011
WBC (x10^3^/µl)	7.1 ± 2.67	7.4 ± 3.37	7.6 ± 3.31	<0.001	7.2 ± 2.87	7.4 ± 3.35	7.2 ± 2.79	0.115
Hemoglobin (g/dL)	12.6 ± 1.8	12.0 ± 1.8	11.7 ± 1.8	<0.001	12.4 ± 1.8	12.0 ± 1.9	11.9 ± 1.7	<0.001
Platelet (x10^3^/µl)	232 ± 75	243 ± 86	247 ± 106	<0.001	236 ± 83	237 ± 88	247 ± 86	0.007
Protein (g/dL)	6.8 ± 0.8	6.7 ± 0.8	6.6 ± 0.8	<0.001	6.8 ± 0.8	6.7 ± 0.8	6.7 ± 0.8	<0.001
Albumin (g/dL)	3.9 ± 0.5	3.8 ± 0.6	3.6 ± 0.6	<0.001	3.9 ± 0.6	3.8 ± 0.6	3.8 ± 0.6	<0.001
Cholesterol (mg/dL)	160 ± 39	161 ± 45	154 ± 41	<0.001	161 ± 42	156 ± 39	157 ± 41	0.003
Alkaline phosphatase (U/L)	89 ± 64	94 ± 78	95 ± 67	0.022	90 ± 68	92 ± 69	98 ± 71	0.019
ALT (U/L)	26 ± 43	25 ± 44	23 ± 31	0.044	27 ± 47	23 ± 28	24 ± 33	0.010
AST (U/L)	33 ± 47	34 ± 56	31 ± 28	0.253	34 ± 54	31 ± 26	31 ± 31	0.108
Creatinine (mg/dL)	0.86 ± 0.32	0.86 ± 0.36	0.85 ± 0.35	0.274	0.86 ± 0.31	0.87 ± 0.37	0.85 ± 0.35	0.460
eGFR (ml/min/1.73 m^2^)	77.4 ± 16.8	75.0 ± 19.1	73.2 ± 19.5	<0.001	76.8 ± 17.1	74.8 ± 19.4	74.4 ± 19.0	<0.001
MMSE-KC (number)	26.6 ± 1.6	21.6 ± 1.1	15.4 ± 3.4	<0.001	24.0 ± 4.4	22.5 ± 5.0	20.6 ± 5.4	<0.001
SGDS-K (number)	3.4 ± 3.5	4.7 ± 3.9	6.0 ± 4.3	<0.001	1.7 ± 1.4	6.6 ± 1.4	11.8 ± 1.6	<0.001

ALT: alanine aminotransferase; AST: aspartate aminotransferase; BMI: body mass index; CG1: MMSE-KC score ≥ 24; CG2: MMSE-KC score 20–23; CG3: MMSE-KC score ≤ 19; CMI: Charlson’s comorbidity index; CVD: cardiovascular disease; DBP: diastolic blood pressure; DG1: SGDS-K score < 5; DG2: SGDS-K score 5–9; DG3: SGDS-K score ≥ 10; DM: diabetes mellitus; eGFR: estimated glomerular filtration rate calculated using the CKD-EPI 2009 equation; HTN: hypertension; MMSE-KC: Mini-Mental State Examination, Korean version of the CERAD assessment packet; SBP: systolic blood pressure; SGDS-K: Short Geriatric Depression Scale, Korean version; WBC: white blood cell count.

*Cancer indicates a history of malignancy identified from electronic health records and includes both current and prior cancer diagnoses.

### Association of MMSE-KC and SGDS-K with RRT and mortality

**Fig 2:**
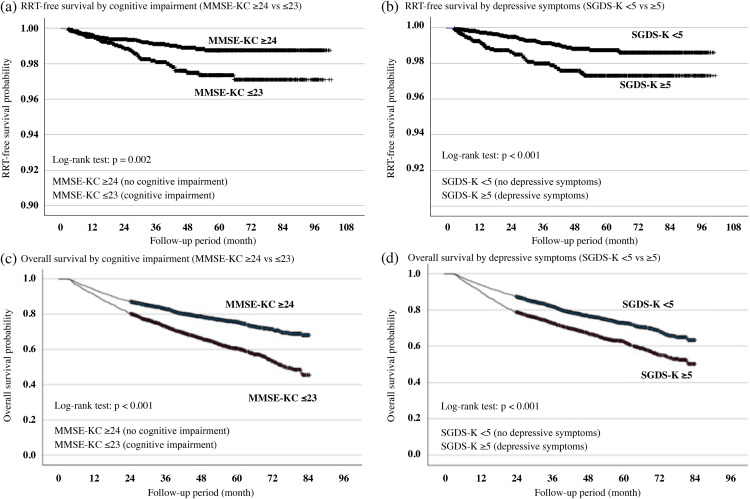
Kaplan–Meier curves for RRT-free and overall survival according to cognitive impairment and depressive symptoms.

Kaplan–Meier survival curves demonstrated significant differences in both RRT-free survival and overall survival based on cognitive impairment and depressive symptoms (log-rank test p-values ranging from <0.001 to 0.002). Participants with cognitive impairment or depressive symptoms had lower survival rates than those with no cognitive impairment or no depressive symptoms. SGDS-K showed a more pronounced separation of RRT-free survival curves compared with MMSE-KC (Fig 2).

**Fig 3:**
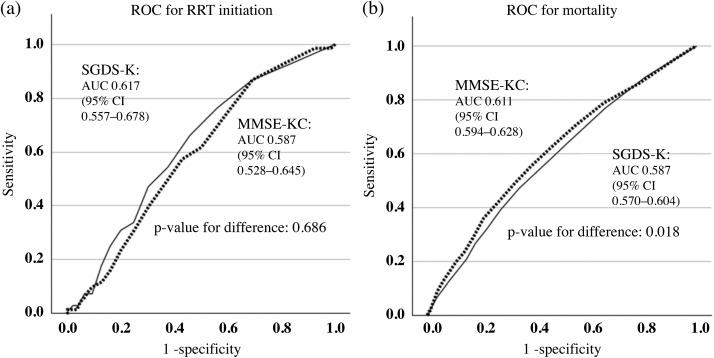
ROC curves for predicting RRT initiation and mortality based on MMSE-KC and SGDS-K scores.

The AUCs for incident RRT were 0.617 (95% CI 0.557–0.678) for SGDS-K and 0.587 (95% CI 0.528–0.645) for MMSE-KC (p = 0.686). For mortality, the AUCs were 0.587 (95% CI 0.570–0.604) and 0.611 (95% CI 0.594–0.628), respectively, indicating a better discriminative ability of MMSE-KC compared with the SGDS-K (p = 0.018) (Fig 3). As a complementary time-dependent measure of discriminative performance, C-indices derived from the Cox models were comparable between the SGDS-K- and MMSE-KC-based models (SGDS-K: C = 0.8390; MMSE-KC: C = 0.8325).

Table 2 summarizes the adjusted associations between cognitive impairment, depressive symptoms, and the risks of incident RRT and mortality. After adjustment for age, sex, baseline eGFR, diabetes mellitus, and hypertension, depressive symptoms (SGDS-K ≥ 5) were associated with a higher risk of incident RRT (HR: 2.066, 95% CI 1.263–3.377, p = 0.004) and mortality (HR: 1.565, 95% CI 1.412–1.735, p < 0.001). Cognitive impairment (MMSE-KC ≤ 23) was also significantly associated with incident RRT (HR: 2.028, 95% CI 1.208–3.405, p = 0.007) and mortality (HR: 1.728, 95% CI 1.550–1.927, p < 0.001).

**Table 2:** Associations of cognitive impairment and depressive symptoms with incident RRT and mortality.

A. For incident RRT					
Variable	B	Wald	HR	95% CI for HR	p-value
SGDS-K (continuous)	0.087	8.464	1.091	1.029–1.156	0.004
Depressive symptoms (SGDS-K ≥ 5)	0.725	8.366	2.066	1.263–3.377	0.004
MMSE-KC (continuous)	−0.055	4.829	0.946	0.901–0.994	0.028
Cognitive impairment (MMSE-KC ≤ 23)	0.707	7.156	2.028	1.208–3.405	0.007
**B. For mortality**					
SGDS-K (continuous)	0.061	92.617	1.062	1.049–1.076	<0.001
Depressive symptoms (SGDS-K ≥ 5)	0.448	72.522	1.565	1.412–1.735	<0.001
MMSE-KC (continuous)	−0.054	116.949	0.947	0.938–0.956	<0.001
Cognitive impairment (MMSE-KC ≤ 23)	0.547	97.124	1.728	1.550–1.927	<0.001

MMSE-KC: Mini-Mental State Examination, Korean version of the CERAD assessment packet; RRT: renal replacement therapy; SGDS-K: Short Geriatric Depression Scale, Korean version.

Cognitive impairment was defined as an MMSE-KC score ≤23, and depressive symptoms were defined as an SGDS-K score ≥5.

Cox proportional hazards models were adjusted for age, sex, baseline eGFR, diabetes mellitus, and hypertension.

Models for incident RRT and mortality both included 5,191 participants, with 68 RRT events and 1,512 deaths, respectively; analyses were conducted using a complete-case approach without imputation.

Regarding additional analyses, we performed Fine–Gray competing-risks regression while treating death as a competing event for incident RRT; 50 deaths occurred prior to RRT initiation in this cohort, and death was accordingly treated as a competing event in the Fine–Gray models. However, the Fine–Gray sub-distribution hazard ratios were similar in direction and magnitude to the Cox estimates (SGDS-K: SHR 1.08, 95% CI 1.02–1.14; MMSE-KC: SHR 0.96, 95% CI 0.92–1.00), indicating that accounting for incident RRT while considering competing mortality did not materially alter the associations of cognitive impairment or depressive symptoms.

Overall, these results indicate that cognitive impairment and depressive symptoms are both associated with increased risks of incident RRT and mortality in this older adult population, with stronger and more consistent associations observed for mortality. In particular, while both conditions were linked to RRT initiation in the overall cohort, subsequent subgroup analyses (Table 3) clarified that the strength of the association with RRT may vary depending on patient characteristics, such as age and baseline kidney function.

**Table 3:** Associations of cognitive impairment and depressive symptoms with outcomes in subgroups.

Group		For incident RRT	HR	95% CI for HR		p-value
Age (years)	< 75	MMSE-KC (≤ 23)	3.489	1.299	9.372	0.013
		SGDS-K (≥ 5)	3.014	1.122	8.096	0.029
	≥ 75	MMSE-KC (≤ 23)	1.638	0.944	2.843	0.079
		SGDS-K (≥ 5)	1.904	1.103	3.286	0.021
Gender	Male	MMSE-KC (≤ 23)	2.125	1.144	3.950	0.017
		SGDS-K (≥ 5)	3.323	1.791	6.163	<0.001
	Female	MMSE-KC (≤ 23)	3.736	1.506	9.268	0.004
		SGDS-K (≥ 5)	1.560	0.733	3.321	0.249
DM	(-)	MMSE-KC (≤ 23)	1.697	0.951	3.029	0.074
		SGDS-K (≥ 5)	1.819	1.020	3.246	0.043
	(+)	MMSE-KC (≤ 23)	3.226	1.261	8.254	0.015
		SGDS-K (≥ 5)	3.049	1.243	7.481	0.015
HTN	(-)	MMSE-KC (≤ 23)	2.881	1.557	5.334	<0.001
		SGDS-K (≥ 5)	2.134	1.181	3.858	0.012
	(+)	MMSE-KC (≤ 23)	1.173	0.525	2.621	0.697
		SGDS-K (≥ 5)	2.191	0.973	4.937	0.058
Cancer	(-)	MMSE-KC (≤ 23)	2.696	1.377	5.276	0.004
		SGDS-K (≥ 5)	2.638	1.364	5.101	0.004
	(+)	MMSE-KC (≤ 23)	1.503	0.732	3.084	0.267
		SGDS-K (≥ 5)	1.639	0.795	3.377	0.190
Charlson’s comorbidity index	< 3	MMSE-KC (≤ 23)	3.635	1.480	8.930	0.005
		SGDS-K (≥ 5)	1.579	0.682	3.656	0.287
	≥ 3	MMSE-KC (≤ 23)	1.530	0.857	2.732	0.150
		SGDS-K (≥ 5)	2.565	1.425	4.615	0.002
eGFR (ml/min/1.73 m^2^)	≥ 75	MMSE-KC (≤ 23)	2.869	1.128	7.300	0.027
		SGDS-K (≥ 5)	2.649	1.065	6.590	0.036
	< 75	MMSE-KC (≤ 23)	1.626	0.925	2.858	0.091
		SGDS-K (≥ 5)	1.893	1.080	3.319	0.026
**Group**		**For mortality**	**HR**	**95% CI for HR**		**p-value**
Age (years)	< 75	MMSE-KC (≤ 23)	1.708	1.362	2.143	<0.001
		SGDS-K (≥ 5)	1.782	1.426	2.227	<0.001
	≥ 75	MMSE-KC (≤ 23)	1.553	1.384	1.743	<0.001
		SGDS-K (≥ 5)	1.392	1.243	1.559	<0.001
Gender	Male	MMSE-KC (≤ 23)	2.022	1.769	2.311	<0.001
		SGDS-K (≥ 5)	1.824	1.594	2.086	<0.001
	Female	MMSE-KC (≤ 23)	2.236	1.899	2.647	<0.001
		SGDS-K (≥ 5)	1.572	1.345	1.836	<0.001
DM	(-)	MMSE-KC (≤ 23)	1.711	1.522	1.924	<0.001
		SGDS-K (≥ 5)	1.549	1.378	1.742	<0.001
	(+)	MMSE-KC (≤ 23)	1.770	1.443	2.172	<0.001
		SGDS-K (≥ 5)	1.440	1.180	1.756	<0.001
HTN	(-)	MMSE-KC (≤ 23)	1.789	1.588	2.016	<0.001
		SGDS-K (≥ 5)	1.521	1.350	1.713	<0.001
	(+)	MMSE-KC (≤ 23)	1.640	1.353	1.988	<0.001
		SGDS-K (≥ 5)	1.569	1.296	1.899	<0.001
Cancer	(-)	MMSE-KC (≤ 23)	2.365	1.987	2.815	<0.001
		SGDS-K (≥ 5)	1.728	1.462	2.042	<0.001
	(+)	MMSE-KC (≤ 23)	1.559	1.374	1.770	<0.001
		SGDS-K (≥ 5)	1.553	1.367	1.765	<0.001
Charlson’s comorbidity index	< 3	MMSE-KC (≤ 23)	2.027	1.742	2.359	<0.001
		SGDS-K (≥ 5)	1.524	1.312	1.772	<0.001
	≥ 3	MMSE-KC (≤ 23)	1.478	1.288	1.695	<0.001
		SGDS-K (≥ 5)	1.558	1.359	1.786	<0.001
eGFR (ml/min/1.73 m^2^)	≥ 75	MMSE-KC (≤ 23)	1.801	1.571	2.063	<0.001
		SGDS-K (≥ 5)	1.537	1.341	1.761	<0.001
	< 75	MMSE-KC (≤ 23)	1.631	1.401	1.900	<0.001
		SGDS-K (≥ 5)	1.518	1.306	1.765	<0.001

CCI, Charlson’s comorbidity index; DM, diabetes mellitus; eGFR, estimated glomerular filtration rate; HTN, hypertension; MMSE-KC, Mini-Mental State Examination (Korean version); RRT, renal replacement therapy; SGDS-K, Short Geriatric Depression Scale (Korean version).

Cognitive impairment was defined as an MMSE-KC score ≤23, and depressive symptoms were defined as an SGDS-K score ≥5.

Hazard ratios (HRs) and 95% confidence intervals (CIs) were estimated using univariate Cox proportional hazards models within each subgroup.

Baseline renal function was stratified at an age-adapted eGFR threshold of 75 mL/min/1.73 m^2^; sensitivity analyses using a 60 mL/min/1.73 m^2^ cutoff are shown in S2 Table. Subgroup analyses were exploratory and unadjusted.

### Impacts of cognitive impairment and depressive symptoms by subgroup

Table 3 presents stratified analyses of cognitive impairment and depressive symptoms with incident RRT and mortality. Subgroup analyses were conducted while stratified by age (< 75 vs ≥ 75 years), sex (male vs female), diabetes mellitus, hypertension, cancer history, Charlson’s comorbidity index (< 3 vs ≥ 3), and baseline renal function using an age-adapted eGFR threshold of 75 mL/min/1.73 m^2^ (≥75 vs < 75), which may better capture functional variability in older adults [15,16]. Sensitivity analyses using the conventional CKD threshold of 60 mL/min/1.73 m^2^ yielded directionally consistent associations and are presented in S2 Table.

Among participants aged < 75 years, both cognitive impairment (MMSE-KC score < 23) and depressive symptoms (SGDS-K score ≥ 5) were associated with an increased risk of incident RRT. In those aged ≥ 75 years, only depressive symptoms remained significantly associated with RRT initiation, as cognitive impairment did not reach statistical significance.

When stratified by baseline renal function, a distinct pattern emerged. Depressive symptoms were significantly associated with incident RRT in both preserved (eGFR ≥ 75 ml/min/1.73 m^2^) and impaired kidney function groups (eGFR < 75), whereas cognitive impairment was associated with incident RRT only among participants with preserved renal function (i.e., non-statistically significant in those with impaired renal function). These findings suggest that the additional prognostic contribution of cognitive impairment to renal outcomes requiring RRT may diminish when baseline kidney function is already substantially reduced.

For mortality, both cognitive impairment and depressive symptoms demonstrated robust associations with an increased risk of death across all subgroup strata (all p < 0.001), indicating that these geriatric syndromes confer mortality risk regardless of age, comorbidity burden, or baseline kidney function.

Regarding other clinical subgroups, cognitive impairment showed attenuated associations with incident RRT among individuals with cancer or without diabetes, whereas depressive symptoms remained more consistently associated with RRT across these strata. Hypertension status and comorbidity burden did not meaningfully modify either association.

Taken together, these subgroup findings suggest that while depressive symptoms consistently relate to adverse renal outcomes requiring RRT, the association of cognitive impairment with RRT risk may diminish in the context of advanced age or reduced kidney function. Meanwhile, both conditions remain strong predictors of mortality across the studied population.

## Discussion

This study found that both cognitive impairment and depressive symptoms were associated with RRT and mortality in the total cohort. Stratified analyses further showed that the association with RRT differed by patient characteristics, with depressive symptoms related to RRT risk across all strata, whereas cognitive impairment was linked to RRT initiation only among individuals with preserved renal function and younger age (<75 years). Cognitive impairment discriminated mortality risk better than depressive symptoms, whereas no significant difference was observed for RRT prediction. Together, these findings suggest the distinct prognostic relevance of cognitive and psychological factors in older adults.

Depressive symptoms showed a more prominent association with incident RRT, whereas cognitive impairment was more clearly related to mortality. Participants with SGDS-K ≥ 5 demonstrated elevated risks of both outcomes, but this association was more pronounced for RRT initiation, supporting the role of depressive burden in renal disease progression. The Kaplan–Meier curves demonstrated both lower RRT-free survival and reduced overall survival among participants with depressive symptoms; these findings are consistent with prior evidence showing that depression in older adults is associated with higher hospitalization rates, functional decline, and mortality [17,18]. Our study extends these observations through our demonstration that depressive symptoms were consistently associated with RRT initiation even after adjustment for renal function and comorbidities. Rather than implying causality, these patterns suggest the relevance of depressive symptoms for specific prognostics, showing that psychosocial and behavioral vulnerability can influence renal outcomes and the importance of routinely assessing depressive burden when evaluating long-term kidney health in older adults.

Cognitive impairment has been consistently associated with higher mortality, whereas its association with incident RRT has been less evident overall. The Kaplan–Meier survival analysis confirmed its association with reduced survival rates, supporting its strong association with mortality rather than renal outcomes requiring RRT. In line with these findings, previous studies have reported that cognitive impairment is related to long-term health deterioration and increased mortality risk [19,20]. Cognitive impairment has also been associated with cerebrovascular diseases, systemic inflammation, neurodegenerative changes, and polypharmacy, all of which may contribute to elevated mortality risk [21]. Additionally, some mechanisms associated with CKD (e.g., vascular injury, white matter lesions, and uremic toxin accumulation) may further contribute to cognitive decline and overall health deterioration [22]. In our cohort, cognitive impairment was associated with incident RRT primarily among adults < 75 years with preserved renal function, suggesting that cognitive vulnerability may influence renal outcomes in earlier disease stages, whereas overall frailty and comorbidity burden may overshadow its effect on RRT initiation in advanced CKD stages [23]. Given that cognitive impairment demonstrated a stronger and more consistent association with mortality than with RRT, the regular assessment of cognitive status may help identify individuals at elevated long-term mortality risk and guide geriatric care planning.

Subgroup analyses by age and baseline renal function further clarified how depressive symptoms and cognitive impairment differentially relate to the risk of incident RRT. Depressive symptoms have been linked to CKD progression, cardiovascular morbidity, and mortality [24], and often coexist with cognitive impairment in patients with CKD, compounding functional decline and risk of adverse outcomes [25]. In this study, depressive symptoms remained significantly associated with incident RRT across age groups and renal function strata, although the association was stronger and more consistent among individuals with preserved renal function. In contrast, cognitive impairment was linked to RRT initiation only in individuals aged < 75 years, suggesting that while cognitive impairment may contribute to disease progression in the early stages, frailty and multiple comorbidities may play a more dominant role in determining renal outcomes in older adults [23]. Combinedly, these subgroup findings suggest that depressive symptoms exert an adverse influence on renal outcomes across a broad clinical spectrum, whereas the contribution of cognitive impairment becomes more apparent when the physiological reserve is still preserved to some extent. Accordingly, early identification and psychosocial interventions for depressive symptoms may help delay renal deterioration and reduce the likelihood of progressing to RRT [25,26]. In contrast, cognitive monitoring may offer the greatest clinical value in individuals with preserved renal function, enabling preventive strategies before frailty and multimorbidity dominate their overall clinical trajectories.

Predictive modeling provided additional context regarding the distinct prognostic relevance of depressive symptoms and cognitive impairment. For mortality, cognitive impairment demonstrated moderately greater discriminative ability than depressive symptoms, whereas their performance for predicting incident RRT was comparable. These findings suggest that cognitive impairment may be more informative for long-term mortality stratification, while depressive symptoms may capture dimensions of vulnerability more relevant to renal deterioration.

To account for time-to-event information, we evaluated model discrimination using Harrell’s C-index derived from Cox proportional hazards models. The C-indices for MMSE-KC– and SGDS-K–based models were similar, indicating overlapping time-dependent discriminative performance. Worthy of note is that although the absolute AUC differences were statistically significant, they were also small. Thus, these distinctions should be interpreted cautiously, as they provide evidence supportive of cognitive impairment and depressive symptoms being relevant for different prognostics rather than delineating the definitive superiority of one measure over the other.

Overall, these results support that depressive symptoms and cognitive impairment contribute to prognosis through partially distinct pathways, with depressive burden being more closely linked to renal outcomes and cognitive decline more strongly aligned with mortality risk over time. In summary, our results support incorporating cognitive and depressive symptom assessments into routine geriatric risk stratification.

## Strengths and limitations

This study has several strengths. The use of a large, well-characterized cohort of older adults enabled a comprehensive analysis of the associations between cognitive impairment, depressive symptoms, and adverse clinical outcomes. In addition to survival analyses and ROC comparisons, time-dependent evaluation using C-indices and competing-risk analyses provided complementary evidence regarding the prognostic relevance of cognitive and psychological factors. Importantly, unlike many CKD-specific cohorts, this study included a broader older adult population with heterogeneous renal function, allowing insights that are more applicable to routine geriatric patients and less constrained to patients with established CKD. The findings that cognitive impairment had greater discriminative ability for mortality while depressive symptoms were more closely associated with RRT initiation suggest that distinguishing cognitive and psychological factors according to outcome may provide clinically meaningful insights for individualized geriatric risk stratification and care planning.

However, several limitations should also be considered. First, cognitive impairment and depressive symptoms were assessed only at a single baseline time point, precluding the evaluation of longitudinal changes. Residual confounding may also persist because variables such as social support, medication adherence, and lifestyle factors were not fully captured. Second, the relatively small number of RRT events may have reduced the statistical power of the analyses, particularly for subgroup analyses. Given the limited number of RRT events, estimates related to RRT initiation may be unstable and should be interpreted as associations rather than definitive predictive effects. Third, RRT initiation was identified using hospital electronic health records without linkage to a national dialysis registry; therefore, RRT initiated at other centers may not have been captured, suggesting the potential under-ascertainment of RRT events. Fourth, renal function strata were based on eGFR rather than clinical CKD staging, which may limit direct comparisons with CKD-specific cohorts. Fifth, depressive symptoms and cognitive impairment may partly reflect underlying frailty or multimorbidity rather than act as isolated predictors, and thus may serve as markers of overall vulnerability in older adults, potentially contributing to the observed associations with RRT initiation and mortality. Finally, these findings may not be fully generalizable to populations with different healthcare systems, socioeconomic backgrounds, or cultural influences on cognitive and mental health.

Despite these limitations, the integration of multiple analytic approaches supports the distinct but complementary prognostic roles of cognitive impairment and depressive symptoms in older adults, underscoring the importance of routine screening and early intervention. Future studies should incorporate longitudinal assessments of cognitive and psychological status, include more granular measures of psychosocial factors, and further examine the role of competing risks in renal outcomes.

## Conclusion

This study demonstrated that depressive symptoms were consistently associated with incident RRT and mortality, whereas cognitive impairment was more strongly linked to mortality. Stratified analyses further indicated that depressive symptoms were associated with RRT initiation across age and renal function strata, whereas cognitive impairment was linked to RRT risk primarily among relatively younger individuals with preserved renal function. Meanwhile, cognitive impairment showed a clearer and more consistent association with mortality than depressive symptoms, aligned with its stronger discriminative performance for survival outcomes.

These findings underline the distinct prognostic implications of depressive symptoms and cognitive impairment in older adults. Depressive symptoms may reflect vulnerability affecting renal outcomes and subsequent treatment needs, while cognitive impairment appears to signify long-term mortality risk. Accordingly, integrating both psychological and cognitive assessments into routine geriatric care may support early risk stratification and individualized management. Interventions targeting depressive symptoms may help delay clinical deterioration and reduce treatment burden, whereas monitoring cognitive decline may aid in identifying individuals at elevated mortality risk. Future studies should evaluate whether tailored psychosocial and cognitive interventions improve long-term outcomes in aging populations.

## Supporting information

S1 Table Completeness of data. (DOCX)

S2 Table Baseline kidney function and sensitivity analyses stratified by eGFR (≥60 vs < 60 ml/min/1.73 m²). (DOCX)
