# Supplementary material for: Cognitive impairment and depressive symptoms to predict renal outcome and mortality in older adult patients
Source: PLoS One. 2026 Mar 2;21(3):e0342924. doi: 10.1371/journal.pone.0342924 (PMC12952636; doi:10.1371/journal.pone.0342924)
Supplement: S1 Table — (DOCX) [file pone.0342924.s001.docx]

|  | **S1 Table.** Completeness of data. | | |  |  |  |
| --- | --- | --- | --- | --- | --- | --- |
|  |  |  |  |  |  |  |
|  |  |  | Parameter | Number | Completeness (%) |  |
|  | AGE |  | Age | 5191 | 100.0 |  |
|  | SEX |  | Gender | 5191 | 100.0 |  |
|  | PE_DBP |  | Diastolic blood pressure | 4695 | 90.4 |  |
|  | PE_SBP |  | Systolic blood pressure | 4695 | 90.4 |  |
|  | TEST_BMI |  | Body mass index | 5168 | 99.6 |  |
|  | TEST2_CHARLSON |  | Charlson's comorbidity index | 5160 | 99.4 |  |
|  | DX_CANCER_TEST |  | History of cancer | 5191 | 100.0 |  |
|  | DX_CVD_TEST |  | History of cardiovascular disease | 5191 | 100.0 |  |
|  | DX_CKD_TEST |  | History of chronic kidney disease | 5191 | 100.0 |  |
|  | DX_HTN_FINAL_TEST |  | History of hypertension | 5191 | 100.0 |  |
|  | DX_DM_FINAL_TEST |  | History of diabetes mellitus | 5191 | 100.0 |  |
|  | DRUG_ACEIARB_TEST |  | Medication of RAS inhibitor | 5191 | 100.0 |  |
|  | DRUG_ANTIHTN_TEST |  | History of anti-hypertensive medication | 5191 | 100.0 |  |
|  | DRUG_DMMED_TEST |  | History of anti-diabetic medication | 5191 | 100.0 |  |
|  | LAB_WBC_14D |  | WBC | 5041 | 97.1 |  |
|  | LAB_HB_14D |  | Hemoglobin | 5075 | 97.8 |  |
|  | LAB_PLT_14D |  | Platelet | 5075 | 97.8 |  |
|  | LAB_ALB_14D |  | Serum albumin | 5167 | 99.5 |  |
|  | LAB_PROTEIN_14D |  | Serum protein | 5102 | 98.3 |  |
|  | LAB_CHOL_14D |  | Serum cholesterol | 4986 | 96.1 |  |
|  | LAB_CA_14D |  | Serum calcium | 5123 | 98.7 |  |
|  | LAB_P_14D |  | Serum phosphate | 5121 | 98.7 |  |
|  | LAB_ALKP_14D |  | Serum alkaline phosphatase | 5100 | 98.2 |  |
|  | LAB_ALT_14D |  | Serum alanine aminotransferase | 5170 | 99.6 |  |
|  | LAB_AST_14D |  | Serum aspartate aminotransferase | 5170 | 99.6 |  |
|  | LAB_NA_14D |  | Serum sodium | 4971 | 95.8 |  |
|  | LAB_K_14D |  | Serum potassium | 4971 | 95.8 |  |
|  | LAB_CL_14D |  | Serum chloride | 4971 | 95.8 |  |
|  | LAB_GFRCKDEPI_14D |  | Estimated glomerular filtration rate | 5191 | 100.0 |  |
|  | TEST2_MMSEKC |  | MMSE-KC | 5191 | 100.0 |  |
|  | TEST2_SGDSK |  | SGDS-K | 5191 | 100.0 |  |
|  | A_RRT01_HOSPITAL |  | Incidence of renal replacement therapy | 5191 | 100.0 |  |
|  | A_DEATH_KOREA |  | Incidence of mortality | 5191 | 100.0 |  |
|  |  |  |  |  |  |  |
|  |  |  |  |  |  |  |
