# Supplementary material for: Cognitive impairment and depressive symptoms to predict renal outcome and mortality in older adult patients
Source: PLoS One. 2026 Mar 2;21(3):e0342924. doi: 10.1371/journal.pone.0342924 (PMC12952636; doi:10.1371/journal.pone.0342924)
Supplement: S2 Table — (DOCX) [file pone.0342924.s002.docx]

| **S2 Table.** Baseline kidney function and sensitivity analyses stratified by eGFR (≥60 vs <60 ml/min/1.73 m²). | | | | | | | |  |  |  |  |  |
| --- | --- | --- | --- | --- | --- | --- | --- | --- | --- | --- | --- | --- |
|  |  |  |  |  |  |  |  |  |  |  |  |  |
| A. Baseline kidney function distribution | |  |  |  |  |  |  |  |  |  |  |  |
| **Baseline eGFR (ml/min/1.73m²)** | **n** | **%** |  |  |  |  |  |  |  |  |  |  |
| ≥ 90 | 1,104 | 21.3 |  |  |  |  |  |  |  |  |  |  |
| 60–89 | 3,125 | 60.2 |  |  |  |  |  |  |  |  |  |  |
| 45–59 | 572 | 11.0 |  |  |  |  |  |  |  |  |  |  |
| 30–44 | 286 | 5.5 |  |  |  |  |  |  |  |  |  |  |
| 15–29 | 104 | 2.0 |  |  |  |  |  |  |  |  |  |  |
| **Total** | **5,191** | **100.0** |  |  |  |  |  |  |  |  |  |  |
|  |  |  |  |  |  |  |  |  |  |  |  |  |
|  |  |  |  |  |  |  |  |  |  |  |  |  |
| B. Sensitivity analysis for incident RRT stratified by baseline eGFR (≥60 vs <60 ml/min/1.73 m²) | | | | | |  |  |  |  |  |  |  |
| **Predictor** | **eGFR group** | **HR** | **95% CI** | **p-value** |  |  |  |  |  |  |  |  |
| MMSE-KC (≤23) | ≥60 | 2.53 | 1.092–5.847 | 0.030 |  |  |  |  |  |  |  |  |
|  | <60 | 1.36 | 0.752–2.442 | 0.312 |  |  |  |  |  |  |  |  |
| SGDS-K (≥5) | ≥60 | 2.46 | 1.076–5.601 | 0.033 |  |  |  |  |  |  |  |  |
|  | <60 | 1.73 | 0.962–3.109 | 0.067 |  |  |  |  |  |  |  |  |
|  |  |  |  |  |  |  |  |  |  |  |  |  |
| MMSE-KC: Mini-Mental State Examination, Korean version of the CERAD assessment packet ; RRT: renal replacement therapy. | | | | | | | | |  |  |  |  |
| SGDS-K: Short Geriatric Depression Scale, Korean version. | | |  |  |  |  |  |  |  |  |  |  |
| Cox proportional hazards models for incident RRT were adjusted for age, sex, baseline eGFR, diabetes mellitus, and hypertension. | | | | | | | | |  |  |  |  |
| Sensitivity analyses were conducted to assess robustness using a conventional CKD threshold and should be interpreted cautiously due to the limited number of RRT events. | | | | | | | | | | | |  |
